# Preliminary study of the growth variability of *Vibrio parahaemolyticus* in simulated gastric digestion fluids

**DOI:** 10.3389/fmicb.2026.1753353

**Published:** 2026-01-29

**Authors:** Zheng’ao Zhang, Qiong Wu, Zilong Zhang, Haoran Du, Yixuan Yang, Wenhui Wei, Yuyang Zhao, Xiaowei Wang, Minglu Zhang

**Affiliations:** 1 State Environmental Protection Key Laboratory of Food Chain Pollution Control, Beijing Technology and Business University, Beijing, China; 2 Shanghai International Travel Healthcare Center (Shanghai Customs Port Outpatient Department), Beijing, China

**Keywords:** induction, PMAxx-qPCR, resuscitation, VBNC state, *Vibrio parahaemolyticus*

## Abstract

*Vibrio parahaemolyticus* may enter the viable but non-culturable (VBNC) state under specific stress conditions such as low temperature and acidic environments. In this study, we simulated gastric fluid digestion of *V. parahaemolyticus* followed by transfer into intestinal fluids, and monitored changes in culturable cell counts, ATP levels, and morphological changes. The objective was to investigate the effects of pH and treatment duration of simulated gastrointestinal fluids on the induction and resuscitation of the VBNC state. Results showed that after 120 min of digestion in gastric fluid at pH 2.5 with added glucose, the lowest number of bacteria were induced into the VBNC state (1.98 × 10^6^ CFU/mL). In contrast, the highest VBNC induction occurred after 60 min of digestion in gastric fluid at pH 4.5 without glucose (1.26 × 10^7^ CFU/mL). When *V. parahaemolyticus* was treated with gastric fluid at pH 4.5 with glucose, followed by 120 min digestion in intestinal fluid, the highest number of viable cells were resuscitated (1.68 × 10^7^ CFU/mL). Moreover, prolonged exposure to intestinal fluid resulted in a greater number of resuscitated cells, accompanied by higher ATP levels compared with post-gastric fluid digestion. Microscopic observations revealed that most cells regained curved morphology, with elongated particle size and shape more similar to those of viable cells. These findings demonstrate that acidic gastric fluid environments can induce *V. parahaemolyticus* into the VBNC state, and that subsequent exposure to intestinal fluid promotes extensive resuscitation. Resuscitated cells released into the environment may pose potential risks to both ecological systems and human health. This study provides important evidence to inform prevention and control strategies for *V. parahaemolyticus*.

## 1 Introduction

*Vibrio parahaemolyticus* is a common foodborne pathogen widely found in seafood, capable of causing gastroenteritis and related illnesses. Under stress conditions such as acidity, nutrient deprivation, low temperature, or ultraviolet irradiation, this bacterium may enter a viable but non-culturable (VBNC) state. In Xu et al. (1982), first noted that the traditional plate count method has limitations in estimating the survival rates of *Escherichia coli* and *Vibrio cholerae* in estuarine and marine environments. A large proportion of these non-culturable cells are actually viable, a state defined as viable but non-culturable. Oliver (2010) defined bacterial cells in the viable but non-culturable (VBNC) state as those incapable of growing under conventional bacterial culture conditions, while they are actually viable and retain metabolic activity. The VBNC state represents a unique survival strategy in which bacteria, under extreme conditions of pH, temperature, or other environmental stressors, undergo a series of physiological and biochemical changes in morphology, cell wall and membrane composition, and metabolism. Although these bacteria cannot be cultured by conventional methods, they retain metabolically active and can regain pathogenicity upon resuscitation (Pan et al., 2020). VBNC *V. parahaemolyticus* may account for unexplained cases of gastroenteritis. Because they cannot be detected by standard culture methods, diagnostic errors or missed detection are possible. This is particularly concerning when antibiotic therapy fails, potentially delaying treatment and endangering patient health. Moreover, Liu et al. (2013) reported that VBNC *V. parahaemolyticus* can horizontally transfer antibiotic resistance genes. Conventional disinfection methods are often insufficient for eliminating VBNC cells or their resistance genes, thereby exacerbating the spread of antimicrobial resistance.

Upon ingested, *V. parahaemolyticus* encounters gastric acid and pepsin, which degrade surface proteins and damage cellular structures. Under acid stress, the bacteria may enter the VBNC state to reduce metabolic activity and survive. Although unculturable in this state, they can potentially recover pathogenicity upon resuscitation in the alkaline intestinal environment (Wang et al., 2020). Pepsin hydrolyzes peptide bonds in cell wall and membrane proteins, destabilizing bacterial structure. Simulated gastric fluid containing pepsin has been shown to increase inactivation efficiency by 20–30% compared with acid alone (Wang et al., 2019). The bactericidal effect of gastric acid depends on both low pH and pepsin activity; however, bacterial acid resistance mechanisms (e.g., membrane remodeling, stress protein expression) and host factors (gastric acid secretion levels, food matrix) can significantly influence survival. Following gastric fluid passage, the intestinal milieu plays a critical role in survival and resuscitation. Intestinal fluids are typically neutral to weakly alkaline (pH 7.0–8.0) and contain bile salts, pancreatic enzymes, and nutrients such as amino acids and glucose—conditions favorable for bacterial resuscitation from the VBNC state. Bile salts act as surfactants that disrupt cell membranes while simultaneously activating quorum-sensing systems, reinitiating metabolic pathways. Neutral pH relieves intracellular acidification, restoring enzymatic activity and promoting recovery to metabolically active states (Yang et al., 2024). Compared with the harsh gastric fluid environment, intestinal fluids provide a “window phase” for resuscitation by supplying nutrients and optimized physicochemical conditions (Zhou et al., 2022). For example, glucose supports ATP replenishment via glycolysis and modulates membrane permeability, further enhancing resuscitation. This capacity for recovery allows VBNC bacteria to colonize the intestine and trigger infections, resulting in delayed gastroenteritis or systemic illness. Without exposure to intestinal fluid, VBNC cells may remain dormant and pose reduced infection risk (Huang et al., 2025; Zhu et al., 2022). Additionally, resuscitated *V. parahaemolyticus* may form biofilms in intestinal fluids, strengthening adhesion to host epithelial cells (Melo et al., 2019). This transition from “cryptic persistence” to “active invasion” highlights the central role of the intestinal environment in pathogenesis.

Therefore, understanding how intestinal fluids drive the resuscitation of VBNC *V. parahaemolyticus* is essential for designing targeted interventions to reduce foodborne infection risk. This study investigated the induction and resuscitation of the VBNC state under simulated gastric and intestinal conditions. The findings provide insights into the gastrointestinal dynamics of *V. parahaemolyticus* survival and resuscitation, offering valuable evidence for its prevention and control in food safety, clinical practice, and public health.

## 2 Materials and methods

### 2.1 Culture of *Vibrio parahaemolyticus*

A standard strain of *Vibrio parahaemolyticus* (ATCC 17802) was used in this study. Twenty milliliters of the bacterial suspension were inoculated into 200 mL of LB broth and incubated in a shaking incubator at 200 rpm and 37 °C for 12 h. The cultures were subsequently stored at 4 °C for further use.

### 2.2 Preparation of simulated digestive fluids

Simulated digestive fluids were prepared according to the formulation described by Singh and Barnard (2016), and filtered through a 0.22 μm membrane. The pH was adjusted using 1 mol/L HCl and 1 mol/L NaOH. Simulated gastric fluids with pH values of 2.5, 3.5, and 4.5 were prepared, and simulated intestinal fluid was prepared at pH 7.0. All solutions were stored at 4 °C and preheated to 37 °C prior to use.

### 2.3 Experimental procedure

Bacterial suspensions were centrifuged (13,000 rpm, 10 min), and the supernatant was discarded. The pellets were resuspended in simulated gastric fluids of different pH values, with glucose supplementation serving as experimental groups, while fluids without glucose served as controls. The suspensions were incubated at 37 °C in a shaking incubator to mimic gastric peristalsis and ensure full contact between gastric fluid and bacterial cells. To simulate the physiological gastric emptying period (2–4 h postprandially) (Edwards et al., 2019), digestion times of 0, 30, 60, and 120 min were selected. After gastric fluid digestion, samples were transferred to simulated intestinal fluid for 120 min of incubation. At the end of digestion, the supernatant was discarded and the pellets were resuspended in pre-cooled PBS to neutralize residual gastric acid and avoid interference with subsequent assays.

### 2.4 Bacterial culture and enumeration

Samples were serially diluted in PBS, and 100 μL of the appropriate dilution was spread onto TSA plates, which were then incubated at 37 °C for 24 h. Each group was tested in triplicate, and the mean ± standard deviation (SD) was calculated. Colony counts were used to determine the number of culturable bacteria.

### 2.5 Flow cytometry (FCM) analysis

Flow cytometry was performed using a NovoCyte instrument (ACEA Biosciences) (Agus et al., 2025). A bacterial viability staining kit (DMAO/PI, Beyotime) was used to prepare single-stain working solutions of DMAO (1000×) and propidium iodide (PI, 1000×) at 100 × concentration. Staining was performed under light-protected conditions. After staining, samples were kept on ice and analyzed within 1 h using the flow cytometer.

### 2.6 PMAxx-qPCR detection

Propidium monoazide (PMA) pretreatment combined with quantitative PCR (qPCR) was employed (Liu et al., 2018). A *V. parahaemolyticus* nucleic acid detection kit (Jiangsu Shuoshi Biotechnology Co., Ltd.) was used. Amplification was conducted on a QuantStudio Q3 real-time PCR system (Thermo Fisher Scientific), and data were analyzed using the manufacturer’s software.

### 2.7 ATP content determination

Bacterial metabolic activity was assessed using an ATP bioluminescence assay (Lee et al., 2025; Robben et al., 2019). ATP levels were measured in both experimental and control groups using an ATP assay kit (Tiangen Biotech, Beijing, China) following the manufacturer’s instructions.

### 2.8 Scanning Electron microscopy (SEM) analysis

Morphological changes of *V. parahaemolyticus* after simulated gastric fluid digestion were examined using a scanning electron microscope (HITACHI SU8010, Japan). A focused high-energy electron beam was applied to scan the sample surface, and microstructural images were captured (Smith, 1999).

### 2.9 Cell membrane permeability analysis

Cell membrane permeability was analyzed using a multiphoton laser confocal microscope (SP8 DIVE, Leica) with SYTO-9/PI dual-fluorescence staining (Chen et al., 2018; Arefev et al., 2021). Multichannel image fusion was applied, and heat-inactivated bacterial suspensions were used as positive controls to validate staining specificity.

### 2.10 Statistical analysis

Data were expressed as mean ± standard deviation (mean ± SD). All experiments were performed with 3 biological replicates (*n* = 3), and parameter estimation was conducted using a 95% confidence interval. Statistical analysis was performed using SPSS software (version 26.0). One-way analysis of variance (ANOVA) followed by Tukey’s *post hoc* test was used to determine the significance of differences among groups. A *p*-value of < 0.05 was considered statistically significant. The effects of pH and time on culturable bacterial counts and particle size were evaluated using two-way ANOVA. Figures were produced by Origin 2024.

## 3 Results and discussion

### 3.1 Effects of different gastric fluid pH conditions on the Culturable count of *Vibrio parahaemolyticus*

**Figure 1:**
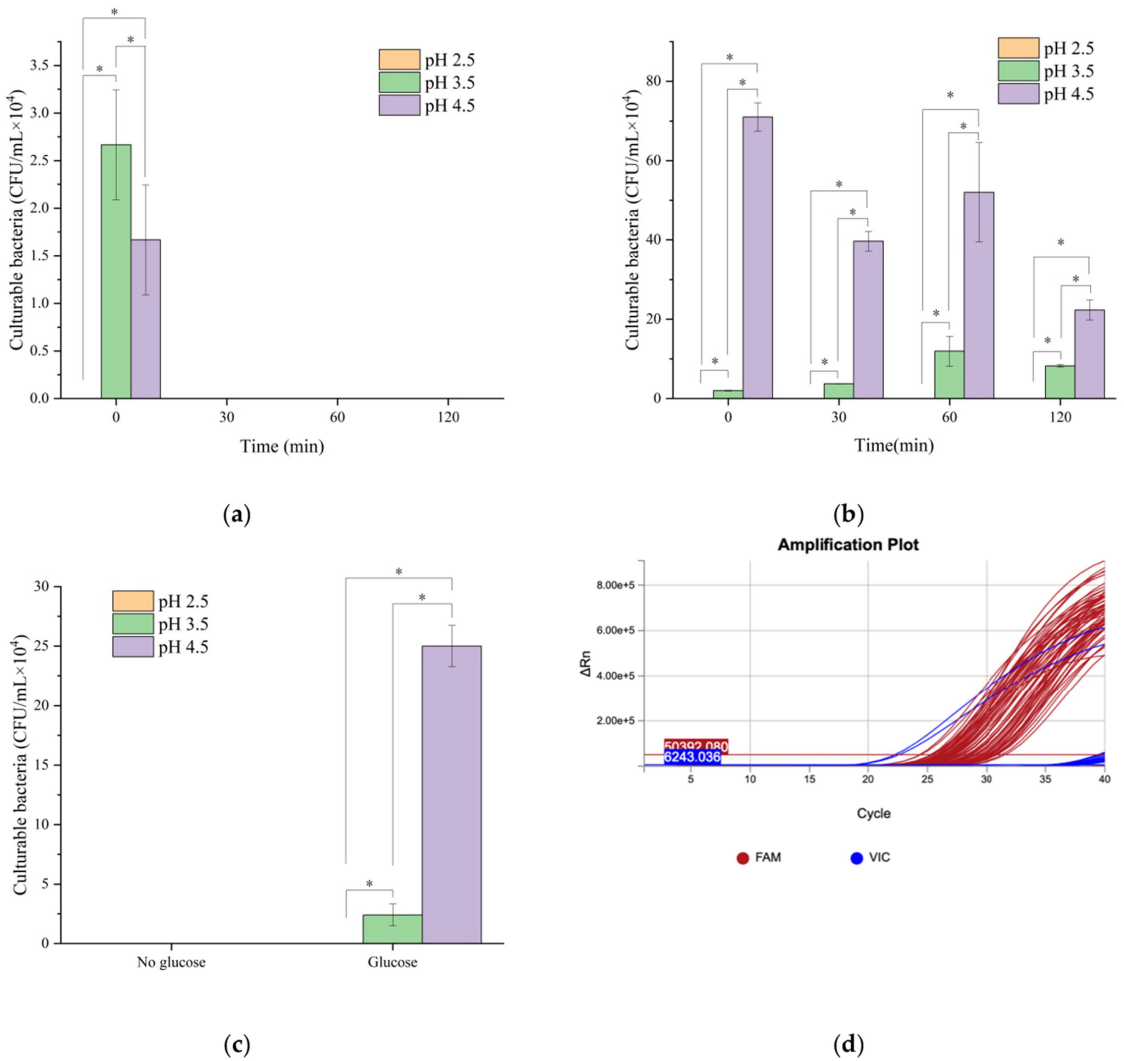
Effects of different pH digestive fluids on the cultivable count of *Vibrio parahaemolyticus*: (**a**) Gastric fluid without glucose; (**b**) Gastric fluid with glucose; (**c**) Intestinal fluid digestion for 120 min; (**d**) PMAxx-PCR amplification curve; *n* = 3, data are presented as mean±SD, *p* < 0.05 indicates significant differences among groups * indicates statistical significance at *p* < 0.05.

Following simulated gastric fluid and intestinal digestion, samples were plated to determine culturable bacterial counts. As shown in Figure 1, gastric fluid pH had a pronounced effect on bacterial survival. In the absence of glucose, culturable counts reached zero after 120 min of gastric fluid digestion under all three pH conditions. However, PMAxx-PCR amplification curves confirmed positive signals in all groups, indicating that a portion of cells had entered the VBNC state (Figure 1a). When glucose was added, culturable counts remained zero after digestion in gastric fluid at pH 2.5, although PMAxx-PCR again revealed entry into the VBNC state (Figure 1b). After digestion at pH 4.5 with glucose, colony counts showed an increasing trend, suggesting that glucose alleviated acid stress by providing energy. At pH 3.5, culturable counts fluctuated downward, possibly due to accumulation of metabolic byproducts or competitive inhibition.

As shown in Figure 1c, once samples transitioned from gastric fluid to intestinal fluid, culturable cells reappeared in the groups pretreated with glucose at pH 3.5 and 4.5, suggesting that VBNC cells were resuscitated under favorable conditions. Consistent with Wang et al. *V. parahaemolyticus* demonstrates limited acid resistance, showing poor survival at pH 2.0–3.0, whereas most of 60 isolates exhibited significant increases in concentration after simulated intestinal digestion. Statistical analysis revealed that pH had a significant effect on culturable counts. *Post hoc* comparisons indicated that counts at pH 2.5 were significantly lower than those at pH 3.5 and 4.5 (*p* < 0.05).

These findings indicate that glucose, as a carbon source, substantially enhanced bacterial metabolic activity. At pH 3.5, although the acidic environment inhibited most microorganisms, the presence of glucose supported greater bacterial survival compared with glucose-free conditions, likely by promoting glycolysis, ATP generation, and enhanced oxidative stress tolerance. Ramesh et al. (2024) reported that glucose supplementation facilitated the resuscitation of two serotypes of *V. parahaemolyticus*, and Hu et al. (2024) successfully used glucose-enriched TSB medium to resuscitate VBNC *Yersinia enterocolitica* (Hu et al., 2024). Together, these results demonstrate that glucose plays a key role in promoting the resuscitation of VBNC bacteria.

### 3.2 Effects of different gastric fluid pH conditions on the proportion of viable *Vibrio parahaemolyticus* cells

**Figure 2:**
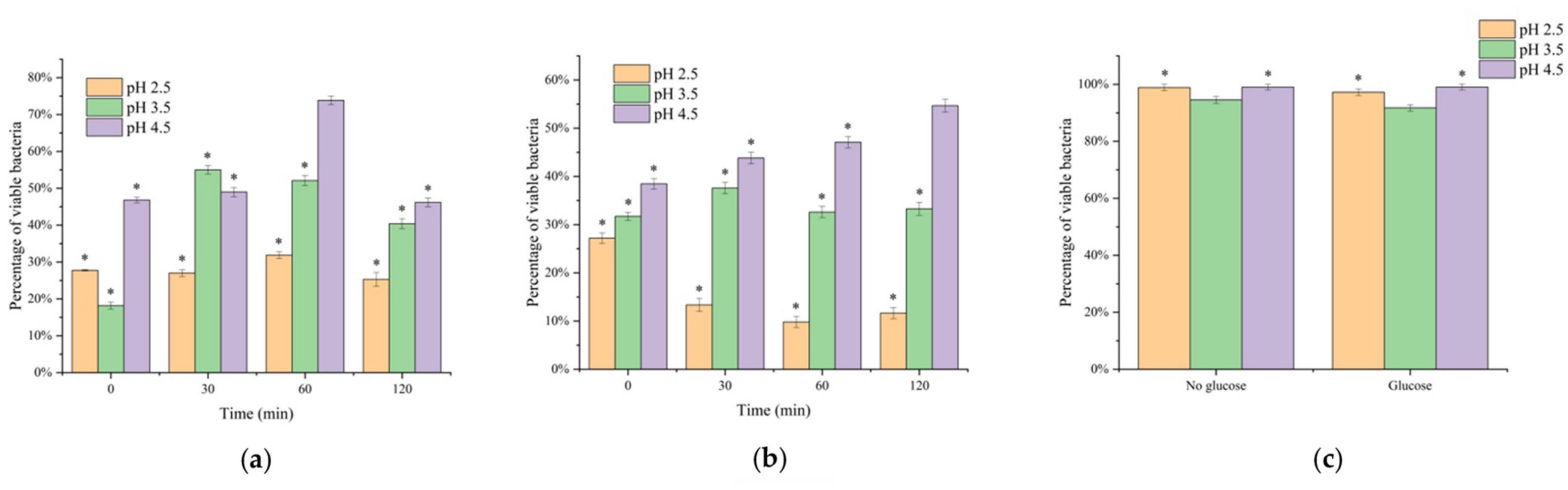
Effects of different pH digestive fluid conditions on the proportion of viable *Vibrio parahaemolyticus*: **(a)** Gastric fluid without glucose; **(b)** Gastric fluid with glucose; **(c)** After intestinal fluid digestion. * indicates statistical significance at *p* < 0.05.

The proportion of viable cells after simulated gastric fluid and intestinal digestion was assessed by flow cytometry. Results showed significant differences in bacterial viability across pH conditions, regardless of glucose supplementation. As shown in Figure 2a, in the glucose-free groups, the proportion of viable cells remained relatively unchanged after digestion in gastric fluid at pH 2.5, but rose to nearly 100% after subsequent intestinal digestion. In contrast, when glucose was added (Figures 2b,c), the viable cell proportions displayed distinct dynamics. After digestion in gastric fluid at pH 2.5, the proportion of viable cells decreased markedly with time. At pH 3.5, viability remained relatively stable, while at pH 4.5 viability continued to increase, ultimately reaching nearly 100% after intestinal digestion.

Integrating these results with plate count data, it is evident that after glucose-free gastric fluid digestion, most bacteria entered the VBNC state but regained viability following intestinal digestion. Conversely, when glucose was present, bacterial growth was more stable, with fewer cells entering the VBNC state. After 120 min of intestinal digestion, nearly all VBNC cells resuscitated, although compared with the glucose-free groups, the proportion of viable cells was lower while dead cells increased (Figure 2a). This suggests that under nutrient-rich conditions, fewer cells transitioned into the VBNC state, maintaining culturability instead. These findings demonstrate that bacterial populations are more likely to enter the VBNC state under extreme stress, whereas nutrient-rich environments favor VBNC resuscitation. Cheng et al. (2023) reported that VBNC cells of lactic acid-induced *Escherichia coli* recovered within 15–18 h in three different nutrient-rich resuscitation media (TSB, Tween80–TSB, and sodium pyruvate–TSB), further supporting the conclusion that adequate nutrients are essential for VBNC recovery.

### 3.3 Effects of simulated digestive fluids on Culturable and VBNC *Vibrio parahaemolyticus*

**Figure 3:**
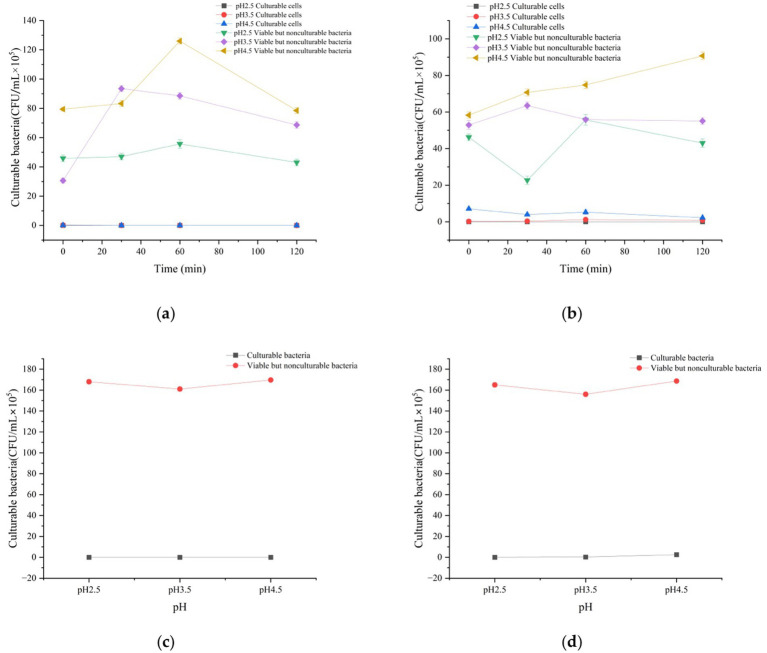
Effects of simulated digestive fluids on the number of cultivable bacteria and VBNC-state bacteria of *Vibrio parahaemolyticus*: **(a)** Gastric fluid without glucose; **(b)** Gastric fluid with glucose; **(c)** After gastric fluid without glucose followed by intestinal fluid digestion; **(d)** After gastric fluid with glucose followed by intestinal fluid digestion. *n* = 3, data are presented as mean ± SD, *p* < 0.05 indicates significant differences among groups.

**Figure 4:**
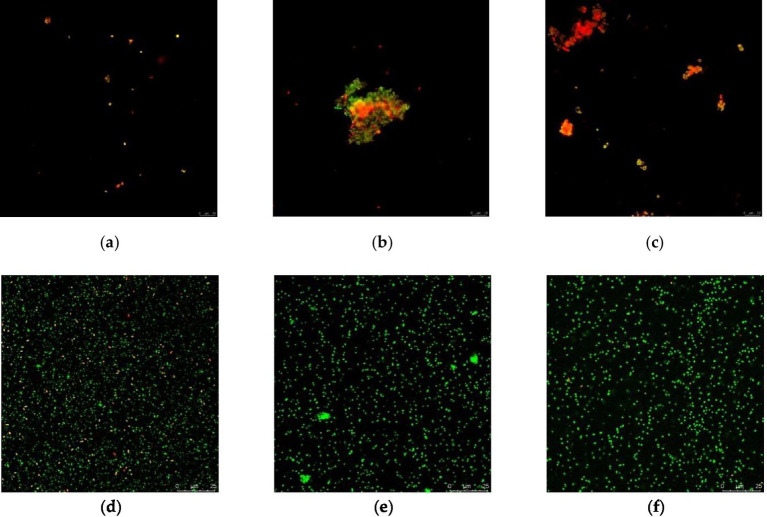
CLSM micrographs: **(a)** Merge image of gastric fluid digestion at pH 2.5 for 120 min; **(b)** Merge image of gastric fluid digestion at pH 3.5 for 120 min; **(c)** Merge image of gastric fluid digestion at pH 4.5 for 120 min; **(d)** Merge image after gastric fluid digestion at pH 2.5 followed by intestinal fluid for 120 min; **(e)** Merge image after gastric fluid digestion at pH 3.5 followed by intestinal fluid for 120 min; **(f)** Merge image after gastric fluid digestion at pH 4.5 followed by intestinal fluid for 120 min.

The number of VBNC cells was estimated from flow cytometry data using the formula: VBNC cell count = (percentage of viable cells × initial bacterial concentration) − culturable count (Baker et al., 2025). A comparison between VBNC and culturable counts is shown in Figure 3. Regardless of glucose supplementation, culturable cells were absent after gastric fluid digestion at pH 2.5. In the absence of glucose, VBNC cell numbers gradually increased and eventually approached the total cell count. By contrast, when glucose was present, VBNC numbers declined rapidly before stabilizing. This is because glucose regulates the energy metabolism mechanism of VBNC: as a carbon source, glucose generates pyruvate through glycolysis, which further enters the TCA cycle to produce a large amount of ATP (Oh et al., 2016). Sufficient ATP can maintain the activity of Na⁺/H⁺-ATPase, pump out excess intracellular H⁺, alleviate intracellular acidification caused by the acidic environment, thereby reducing VBNC induction. Meanwhile, ATP provides energy for cell membrane repair (e.g., phospholipid synthesis), reducing the damage to membrane integrity by gastric acid (CLSM results show a higher proportion of green fluorescence in the glucose group, Figure 4e), and promoting VBNC resuscitation. These findings indicate that strong acid stress markedly suppresses bacterial survival and forces most cells into the VBNC state, whereas glucose alleviates acid stress and reduces the rate of VBNC conversion (Zhang et al., 2025).

At pH 3.5 without glucose, culturable counts were nearly zero, while VBNC numbers initially increased and then stabilized, suggesting that moderate acid stress permitted limited culturability. As shown in Figure 3b, when glucose was added at pH 3.5, culturable counts remained slightly higher and VBNC counts rose only modestly. At pH 4.5 without glucose, culturable counts initially increased slightly but subsequently declined, with a corresponding rise in VBNC counts. Even under near-neutral conditions, bacteria lost culturability rapidly when glucose was absent, likely due to carbon source depletion and energy exhaustion. In contrast, at pH 4.5 with glucose, culturable counts showed a transient early increase before decreasing. Across both pH 3.5 and 4.5 conditions, VBNC numbers rose only moderately, indicating that the presence of glucose under near-neutral pH enabled *V. parahaemolyticus* to sustain metabolic activity and culturability. These results suggest that gastric fluid at pH 4.5 with glucose represents an optimal survival condition, in which metabolic adaptation effectively mitigates acid stress and significantly reduces the transition into the VBNC state (Figures 3a,b).

**Figure 5:**
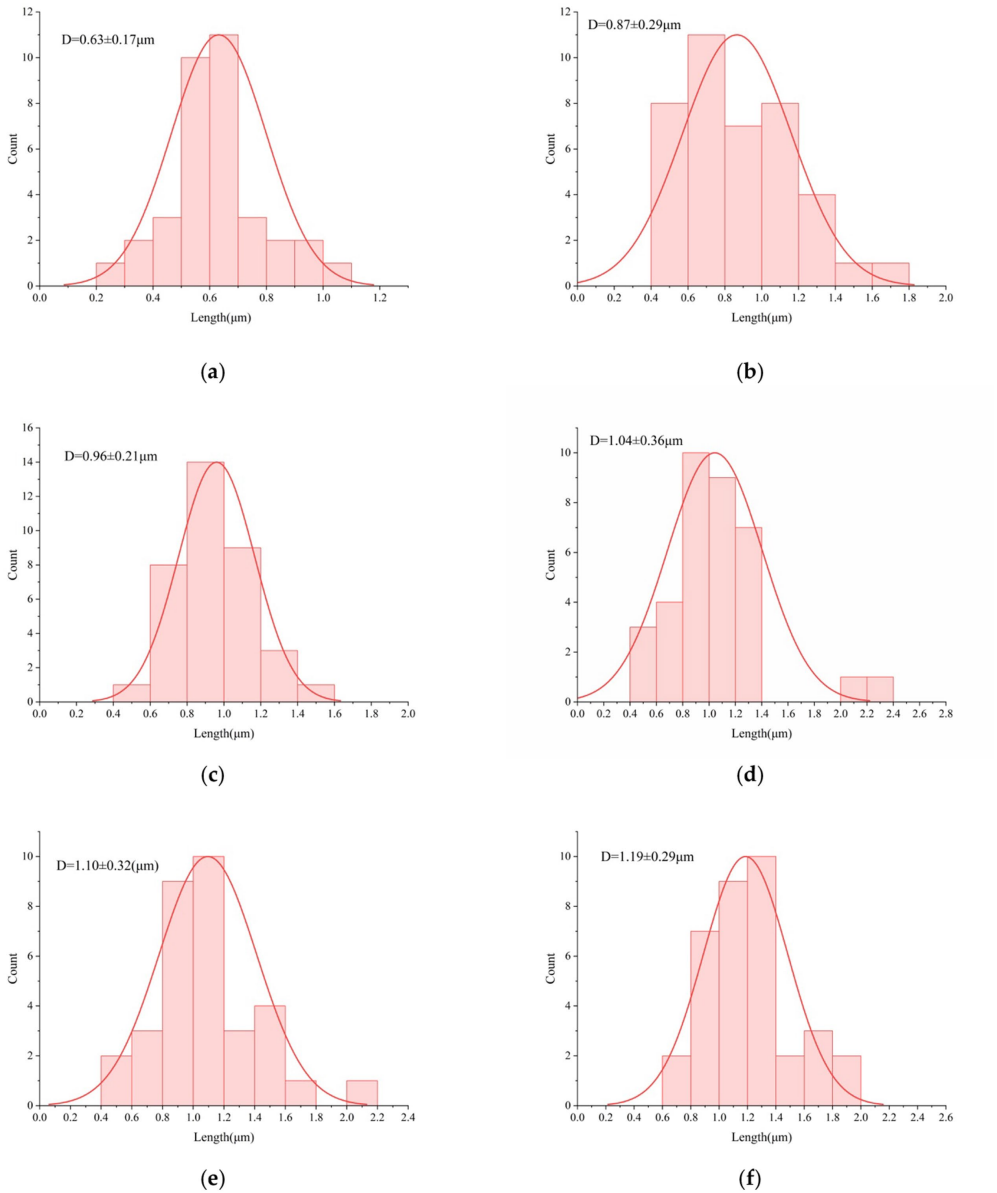
Particle size distribution: **(a)** Gastric fluid digestion at pH 2.5 for 120 min; **(b)** After gastric fluid digestion at pH 2.5 followed by intestinal fluid for 120 min; **(c)** Gastric fluid digestion at pH 3.5 for 120 min; **(d)** After gastric fluid digestion at pH 3.5 followed by intestinal fluid for 120 min; **(e)** Gastric fluid digestion at pH 4.5 for 120 min; **(F)** After gastric fluid digestion at pH 4.5 followed by intestinal fluid for 120 min. *n* = 3, data are presented as mean ± SD, *p* < 0.05 indicates significant differences among groups.

As illustrated in Figures 3c,d, whether or not glucose was present, the number of VBNC cells after gastric fluid digestion far exceeded the number of culturable cells. Following intestinal digestion, culturable counts increased relative to post-gastric fluid digestion levels, indicating that *V. parahaemolyticus* exhibits substantial tolerance to bile salts. Intestinal fluids provided a favorable environment in which most VBNC cells resuscitated. Intestinal fluids provide a favorable environment for VBNC resuscitation via multiple pathways: the neutral pH (7.0) of intestinal fluid alleviates intracellular acidification and restores enzyme activity; bile salts activate the quorum-sensing system to restart metabolic pathways (Yang et al., 2024); amino acids and glucose in the intestine further supplement energy, promoting VBNC cells to recover the curved rod-shaped morphology (SEM Figure 5b,d,f) and culturability. Once excreted into the environment, such resuscitated bacteria may pose considerable ecological and health risks. Previous studies support these findings: Ma et al. (2023) reported that *Escherichia coli* retained high viability after 4 h of exposure to artificial intestinal fluid; Ma and Liu (2019) observed that *Salmonella typhi* not only survived but also adapted and colonized under intestinal conditions; and Fan et al. (2018) demonstrated that *Lactobacillus plantarum* TD109 showed strong bile salt tolerance, with a survival rate of 47.66% after 8 h in simulated intestinal fluid. Collectively, these studies indicate that many bacteria possess inherent bile salt resistance, and that intestinal fluids generally provide more favorable conditions for bacterial growth and VBNC resuscitation compared with gastric fluids.

These findings also highlight the role of glucose as an energy source. Through glycolysis and the tricarboxylic acid (TCA) cycle, glucose metabolism generates ATP to maintain proton pump activity and membrane stability, thereby reducing acid-induced membrane damage and enhancing intracellular pH homeostasis. Furthermore, alkaline byproducts of glucose metabolism (e.g., NH₃) can partially neutralize intracellular H⁺, mitigating acid stress (Oh et al., 2016). Consequently, glucose supplementation resulted in higher proportions of culturable cells, fewer cells transitioning into the VBNC state, and a greater proportion of VBNC cells successfully resuscitating.

### 3.4 Metabolic activity of *Vibrio parahaemolyticus*

**Figure 6:**
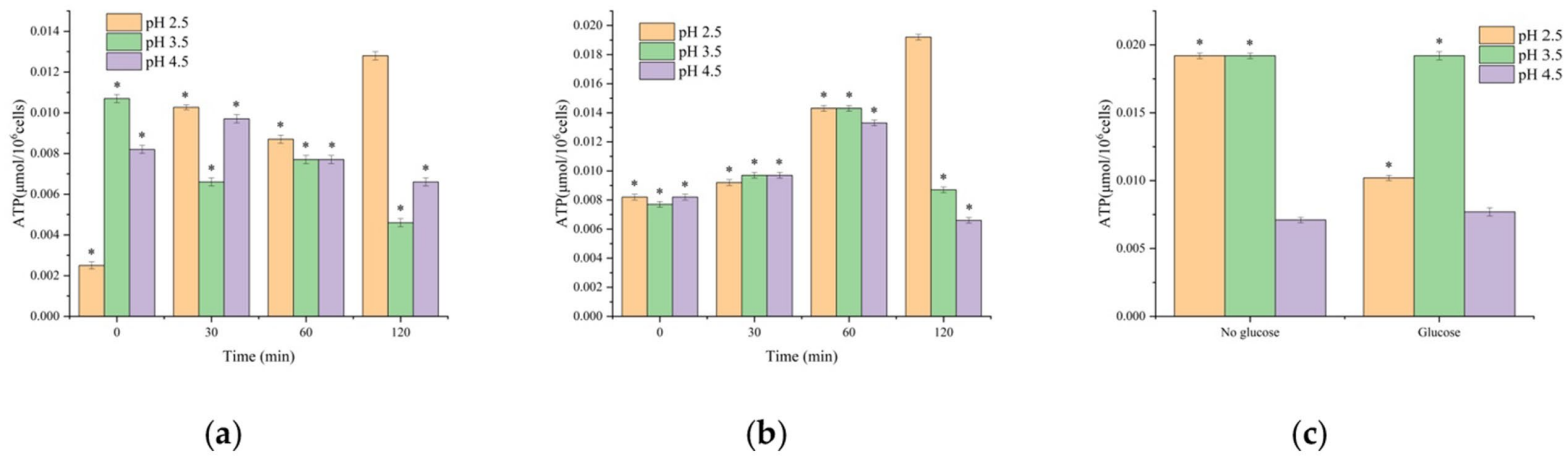
Effects of different digestive fluid conditions on the ATP content of *Vibrio parahaemolyticus*: **(a)** Gastric fluid without glucose; **(b)** Gastric fluid with glucose; **(c)** After intestinal fluid digestion. *n* = 3, data are presented as mean ± SD, *p* < 0.05 indicates significant differences among groups. * indicates statistical significance at *p* < 0.05.

Adenosine triphosphate (ATP) is the central molecule of cellular energy metabolism, and its concentration directly reflects metabolic activity and viability. As shown in Figure 6a, in the absence of glucose, ATP levels declined in a fluctuating manner following gastric fluid digestion, reaching the lowest point at 120 min. The core reasons for the fluctuations in ATP data are related to the metabolic dynamics of cells under different stress conditions: the strongly acidic environment causes a transient increase in cell membrane permeability (supported by CLSM results, Figure 4a), leading to the leakage of intracellular ATP. Meanwhile, glucose metabolism is inhibited (the activity of key glycolytic enzymes decreases), resulting in insufficient ATP synthesis, which is manifested as increased fluctuations. Combined with electron microscopy observations, membrane damage was evident, with some cells entering irreversible death or the VBNC state. Under glucose-free gastric fluid conditions, culturable counts were nearly zero, indicating that *V. parahaemolyticus* could not maintain energy metabolism in an acidic environment. The declining ATP trend corresponded with reduced culturability, suggesting that survival was highly dependent on exogenous energy sources, and that inappropriate acidic conditions drove cells to death or into the VBNC state. In contrast, when glucose was added, ATP levels showed a slow increase within the first 0–60 min. The early increase (0–60 min) in the presence of glucose is attributed to glucose rapidly providing energy through glycolysis to promote ATP synthesis (Oh et al., 2016). After 60 min, the fluctuations tend to stabilize, which may be related to the accumulation of metabolic byproducts (e.g., lactic acid) under acidic conditions that inhibit energy metabolism. Additionally, the influence of the VBNC transition stage: some cells are in the transition state of “viable but non-culturable,” and their metabolism is not completely stagnant (the ATP level is still about 80% of that in normal cells, Wang et al., 2020), leading to a dynamic balance between ATP synthesis and consumption, which is reflected in data fluctuations. After 60 min of gastric fluid digestion, ATP levels at pH 2.5, pH 3.5, and pH 4.5 reached 0.0143, 0.0143, and 0.0133, respectively—substantially higher than the corresponding glucose-free groups (0.0087, 0.0077, 0.0077). At 120 min, ATP levels converged with those of the glucose-free groups, indicating that glucose was efficiently utilized at near-neutral pH (Figures 6a,b). Following pH 4.5 gastric fluid digestion, ATP fluctuations were minimal regardless of glucose supplementation. Together with plate counts and flow cytometry data, this suggests fewer dead cells and only minor differences in ATP between VBNC and viable populations, implying sustained cellular activity. ATP measurements thus provide insight into survival strategies under environmental stress. High ATP levels indicate active energy synthesis, whereas low ATP levels suggest metabolic inhibition, energy depletion, or death. Wang et al. (2020) reported that ATP synthesis declined in VBNC *V. parahaemolyticus* cells, yet intracellular ATP levels still remained ~82.35% of those in normal cells; Hu et al. (2024) found that ATP levels in VBNC *Yersinia enterocolitica* CMCC52225 cells decreased significantly but remained relatively high. Similarly, Bai et al. observed that ATP levels in VBNC *Staphylococcus aureus* cells were approximately two-thirds of those in non-induced cells (Bai et al., 2019). These findings confirm that VBNC bacteria maintain relatively high ATP content, reflecting persistent metabolic activity. In the pH 3.5 glucose-supplemented group, ATP peaked at more than twice the baseline, indicating that glucose alleviated acid-induced metabolic inhibition by providing exogenous energy. This suggests that pH 3.5 allows a balance between acid stress and metabolic efficiency, with glucose utilization most effective at this condition. Conversely, at pH 2.5, large ATP fluctuations were observed regardless of glucose supplementation, suggesting that this represents the tolerance limit for *V. parahaemolyticus*. The instability may be related to membrane permeability loss or inactivation of critical enzymes.

As shown in Figure 6c, ATP levels increased substantially in intestinally digested samples, particularly in groups pretreated with gastric fluids at pH 2.5 and pH 3.5 without glucose, indicating massive resuscitation under intestinal conditions. In the glucose-supplemented groups, ATP was highest following gastric fluid digestion at pH 3.5, reaching 0.0192, further confirming that cells entered the intestinal environment primed for resuscitation. Correlation with VBNC counts indicated that at pH 3.5, regardless of glucose addition, VBNC cell numbers were lowest and ATP levels were highest. This suggests that pH 3.5 conditions cause less cellular damage, facilitating resuscitation of VBNC cells in the intestine.

### 3.5 Morphological changes of *Vibrio parahaemolyticus*

Scanning electron microscopy (SEM) was employed to observe changes in cell length under simulated digestive conditions. SEM images of glucose-supplemented groups exposed to gastric fluids of varying pH revealed that the simulated gastrointestinal environment significantly influenced morphology, VBNC induction, and resuscitation. As shown in Figure 5a, after 120 min of gastric fluid digestion at pH 2.5, cells transformed from curved rods into spherical forms, with mean length shortened to 0.63 ± 0.17 μm (*p* < 0.05). The size distribution shifted from narrow to broad and dispersed, with peaks flattened and shifted leftward, indicating that many cells entered the VBNC state under severe stress (cell shrinkage) or suffered irreversible damage leading to death. In contrast, digestion at pH 3.5 and pH 4.5 yielded mean lengths of 0.96 ± 0.21 μm (*p* < 0.05) (Figure 5c) and 1.10 ± 0.32 μm (*p* < 0.05) (Figure 5e), respectively. These results demonstrate that higher pH environments allowed cells to elongate significantly, with maximum lengths observed at pH 4.5. Thus, lower pH caused more severe structural damage, consistent with stress-induced morphological changes, where many cells transitioned to the VBNC state characterized by shrinkage, membrane disruption, and aggregation into larger clusters. Two-way ANOVA confirmed that both pH and time significantly influenced particle size, with a significant interaction effect (*p* < 0.05).

Figures 5b,d,f illustrate that after subsequent 120 min intestinal digestion, most cells across all groups regained curved morphology. Cell sizes elongated and more closely resembled those of the original inoculum, further demonstrating bile salt tolerance and successful resuscitation under intestinal conditions. These observations confirm that cells induced into the VBNC state by gastric fluid conditions can recover culturability in favorable intestinal environments.

These results align with classical morphological features of VBNC cells: reduced size, surface condensation, and heterogeneous distribution. Prolonged acid stress efficiently induced VBNC states characterized by pore formation, increased adhesion, and enhanced size heterogeneity. Ramesh et al. (2024) reported that VBNC cells consistently adopted spherical morphology, indicating loss of permeability in the dormant state, while culturable cells retained typical curved rod shapes. The most pronounced morphological change in non-culturable cells was the transition from rod-shaped to coccoid clusters. Importantly, VBNC cells in intestinal fluid were observed to resuscitate, regaining curved morphology though often shorter than original cells. Taken together, these findings provide critical morphological evidence that prolonged acidic stress strongly induces VBNC formation in *V. parahaemolyticus*. Key markers include cell shrinkage, heterogeneous size distribution, and surface condensation. Upon transfer to suitable environments, VBNC cells resuscitate, shifting from spherical back to curved rod morphology. This morphological plasticity reflects an essential adaptive strategy for survival under gastrointestinal stress.

### 3.6 Cell membrane permeability of *Vibrio parahaemolyticus*

To investigate the membrane permeability of VBNC cells, confocal laser scanning microscopy (CLSM) was employed. As shown in Figure 4, green fluorescence indicates intact membranes, while red fluorescence indicates membrane damage. Under all conditions, the proportion of dead cells (red fluorescence) gradually increased with digestion time up to 120 min (Figures 4a-c), consistent with plate count results showing culturability approaching zero. However, flow cytometry revealed that even when culturable cells were nearly absent, viable cells were still detected, and their proportion increased with pH. This indicates that many bacteria had entered the VBNC state with markedly restricted metabolic activity. Although glucose supplementation elevated ATP content by supporting metabolism, prolonged digestion led to accumulation of metabolic byproducts or oxidative stress, ultimately compromising membrane integrity (with significantly increased red fluorescence after 120 min). Even with glucose supplementation, long-term exposure to acidic conditions exacerbated membrane damage, suggesting that acid stress irreversibly disrupts membrane proteins and enzymatic activity. Strongly acidic conditions directly compromise membrane integrity, induce proton influx, disrupt intracellular pH homeostasis, and inhibit key enzymes such as ATP synthase. This “intact membrane but arrested metabolism” phenomenon explains the limitations of conventional culture methods (Lin et al., 2024) and highlights the potential for VBNC bacteria to resuscitate in the intestine under bile salt and neutral pH conditions, regaining pathogenicity. Ahire et al. (2022) reported that strains in the VBNC state could resuscitate into a culturable state under simulated colonic conditions. Furthermore, glucose was shown to protect and enhance bacterial viability throughout gastrointestinal transit. Cell membrane permeability analysis further clarified survival states under different gastric fluid conditions. After subsequent intestinal digestion, VBNC cells resuscitated and proliferated under favorable conditions. In groups pretreated with gastric fluids at pH 3.5 and 4.5, intestinal digestion resulted in a high proportion of green fluorescence (Figures 4d–f), indicating relatively minor damage compared with pH 2.5 digestion. Fewer dead cells remained, and more viable cells were resuscitated under intestinal conditions. This phenomenon is closely related to the mechanism by which gastric acid induces the VBNC state: gastric acid damages the lipid bilayer structure of the cell membrane (SEM shows cell shrinkage and membrane breakage, Figure 5A), leading to the leakage of intracellular substances. Meanwhile, acid stress activates the SOS response system of bacteria, upregulates the expression of stress proteins (DnaK, GroEL), inhibits cell division, and promotes cells to enter the VBNC state with low metabolic levels (Pan et al., 2020).

## 4 Conclusion

This study examined the induction of *Vibrio parahaemolyticus* into the VBNC state and its resuscitation under simulated digestive conditions. The results demonstrate that acidic gastric fluid environments strongly induce VBNC formation, whereas intestinal environments promote resuscitation. Without glucose, gastric fluid digestion at pH 2.5 caused VBNC counts to gradually increase while maintaining relatively high ATP levels. At pH 3.5, VBNC counts first increased and then stabilized, indicating moderate acid stress where some cells remained culturable. At pH 4.5, VBNC counts rose slightly but then declined overall, likely due to carbon source depletion, leading to energy exhaustion and lower ATP levels. Glucose supplementation alleviated acid stress by providing exogenous energy and promoting ATP synthesis. However, gastric fluid digestion still resulted in accumulation of metabolic byproducts and oxidative stress, eventually compromising membrane integrity. Morphological observations revealed hallmark VBNC features, including reduced cell size, condensed surfaces, dispersed distributions, and transformation from curved rods to coccoid forms. Under pH 4.5 with glucose, VBNC cell counts were the highest, and after extended intestinal digestion, the largest number of viable cells resuscitated. Morphological recovery was evident, with most cells regaining curved rod shapes and lengths approaching those of actively growing cells. This further confirmed that intestinal fluids provide favorable conditions for VBNC resuscitation. Collectively, this study systematically elucidates the transition of *V. parahaemolyticus* into the VBNC state under acidic stress and its potential resuscitation under suitable conditions. These findings highlight the ecological and public health risks posed by VBNC bacteria and provide theoretical support for developing strategies to control *V. parahaemolyticus* in food safety and human health contexts.

## Data Availability

The original contributions presented in the study are included in the article/supplementary material, further inquiries can be directed to the corresponding authors.
